# Supplementary material for: The Relationship between Emotional Intelligence, Obesity and Eating Disorder in Children and Adolescents: A Systematic Mapping Review
Source: Int J Environ Res Public Health. 2021 Feb 20;18(4):2054. doi: 10.3390/ijerph18042054 (PMC7923289; doi:10.3390/ijerph18042054)
Supplement: Supplementary file 1 [file ijerph-18-02054-s001.pdf]

International Journal of *Environmental Research and Public Health*

MDPI

Supplementary Table S1. Results of the methodological quality assessment the Appraisal tool for Cross-Sectional Studies (AXIS).

		Amado Alonso, 2020	Cuesta-Zamora, 2018	Markey, 2007	Peres, 2017	Pollatos, 2020	Zavala Berbena, 2018	Li, 2019	Li, 2018	Wong, 2014
	**Study design**									
1	Were the aims/objectives of the study clear?	+	+	+	+	+	+	+	+	+
2	Was the study design appropriate for the stated aim(s)?	+	+	-	+	+	+	-	-	+
	**Selection bias**									
3	Was the sample size justified?	+	+	+	-	+	-	-	-	+
4	Was the target/reference population clearly defined? (Is it clear who the research was about?)	+	+	-	+	+	+	+	+	+
5	Was the sample frame taken from an appropriate population base so that it closely represented the target/reference population under investigation?	+	+	-	-	+	+	+	+	+
6	Was the selection process likely to select subjects/participants that were representative of the target/reference population under investigation?	+	-	-	-	+	+	+	+	+
7	Were measures undertaken to address and categorise non-responders?	?	+	?	+	+	?	-	-	+
	**Measurement bias**									
8	Were the risk factor and outcome variables measured appropriate to the aims of the study?	+	+	+	+	+	+	+	+	+
9	Were the risk factor and outcome variables measured correctly using instruments/measurements that had been trialled, piloted or published previously?	+	+	+	+	+	+	+	+	+
	**Reporting bias**									
10	Is it clear what was used to determined statistical significance and/or precision estimates? (e.g. p-values, confidence intervals)	+	+	+	+	+	+	+	+	+
11	Were the methods (including statistical methods) sufficiently described to enable them to be repeated?	+	+	+	+	+	+	+	+	+
12	Were the basic data adequately described?	+	+	+	+	+	+	-	+	+
13	Does the response rate raise concerns about non-response bias?	?	-	+	?	+	?	+	+	+
14	If appropriate, was information about non-responders described?	?	+	+	?	+	?	+	+	+
15	Were the results internally consistent?	+	+	+	+	+	+	+	+	+
16	Were the results presented for all the analyses described in the methods?	+	+	+	-	+	+	+	+	+

	**Confounding**									
17	Were the authors' discussions and conclusions justified by the results?	+	+	+	-	+	+	+	+	-
18	Were the limitations of the study discussed?	+	+	+	+	+	+	+	+	+
19	Were there any funding sources or conflicts of interest that may affect the authors' interpretation of the results?	+	+	+	+	+	+	+	+	+
20	Was ethical approval or consent of participants attained?	+	+	+	+	+	+	+	+	+

Note. Plus signs (+) indicate absence of risk of bias, minus signs (-) indicate potential risk of bias and question marks (?) indicate absence of information to assess the AXIS item.
